# TCF7L2 polymorphisms and inflammatory markers before and after treatment with fenofibrate

**DOI:** 10.1186/1758-5996-1-16

**Published:** 2009-10-12

**Authors:** Edmond K Kabagambe, Stephen P Glasser, Jose M Ordovas, Daruneewan Warodomwichit, Michael Y Tsai, Paul N Hopkins, Ingrid B Borecki, Mary Wojczynski, Donna K Arnett

**Affiliations:** 1 Department of Epidemiology, School of Public Health and Clinical Nutrition Research Center, University of Alabama, Birmingham, AL 35294, USA; 2 Division of Preventive Medicine, Department of Medicine, University of Alabama, Birmingham, AL 35294, USA; 3 Nutrition and Genomics Laboratory, JM-USDA Human Nutrition Research Center on Aging, Tufts University, Boston, MA 02111, USA; 4 Department of Medicine, Ramathibodi Hospital, Mahidol University, Bangkok 10400 Thailand; 5 Department of Laboratory Medicine and Pathology, University of Minnesota, 420 Delaware Street SE, Minneapolis, MN 55455, USA; 6 Department of Internal Medicine, University of Utah, 410 Chipeta Way, Salt Lake City, UT 84108, USA; 7 Division of Statistical Genomics, Department of Genetics, Washington University, School of Medicine, 4444 Forest Park Boulevard - Box 8506, St Louis, MO 63108, USA; 8 Department of Biostatistics, Section on Statistical Genetics, University of Alabama, Birmingham, AL, 35294, USA

## Abstract

**Background:**

Inflammation is implicated in causing diabetes. We tested whether transcription factor 7 like-2 (TCF7L2) gene polymorphisms (rs12255372 and rs7903146), consistently associated with type 2 diabetes, are associated with plasma concentrations of inflammatory markers before and after three weeks of daily treatment with fenofibrate.

**Methods:**

Men and women in the Genetics of Lipid-Lowering Drugs and Diet Network study (n = 1025, age 49 ± 16 y) were included. All participants suspended use of lipid-lowering drugs for three weeks and were then given 160 mg/day of fenofibrate for three weeks. Inflammatory markers and lipids were measured before and after fenofibrate. ANOVA was used to test for differences across TCF7L2 genotypes.

**Results:**

Under the additive or dominant model, there were no significant differences (*P *> 0.05) in the concentrations of inflammatory markers (hsCRP, IL-2, IL-6, TNF-α and MCP-1) across TCF7L2 genotypes in the period before or after treatment. For both rs12255372 and rs7903146, homozygote T-allele carriers had significantly higher (*P *< 0.05) post-fenofibrate concentrations of MCP-1 in the recessive model. No other significant associations were detected.

**Conclusion:**

Overall these data show no association between TCF7L2 polymorphisms and the inflammatory markers suggesting that the effects of TCF7L2 on diabetes may not be via inflammation.

## Background

Two polymorphisms (rs12255372 and rs7903146) in the transcription factor 7 like-2 (TCF7L2) gene have been consistently associated with diabetes in various populations [1-3]. Diabetes is an inflammatory condition in which up-regulation of inflammatory markers such as tumor necrosis factor (TNF)-α leads to impaired insulin signaling [4]. *In vitro*, TNF-α has been shown to enhance the transcriptional activity of TCF7L2 leading to reduced adipogenesis [5,6]. However, the sequence of events relating inflammation and TCF7L2 activity *in vivo *is not well understood. There are no published reports on whether the biological effects of TCF7L2 gene on diabetes are partly via inflammation or whether inflammation alters the effects of TCF7L2 on insulin secretion and other metabolic traits. Moreover how the potential association between TCF7L2 and inflammatory markers may be modified by diet and other environmental exposures such as fenofibrate is not known.

## Materials and methods

The subjects were 1025 Caucasian men and women in the Genetics of Lipid-Lowering Drugs and Diet Network (GOLDN) family study with three-generational pedigrees in two genetically homogeneous centers in Minneapolis, MN and Salt Lake City, Utah [7]. The GOLDN study is part of the PROgram for GENetic Interaction (PROGENI) Network, a group of National Institutes of Health (NIH)-funded family intervention studies focusing on gene-environment interactions. The main aim of the GOLDN study is to characterize the genetic basis of the variable response of triglycerides to two environmental contexts, one that raises triglycerides (dietary fat), and one that lowers triglycerides (fenofibrate treatment). Men and women in this study participated in a three week open-label clinical trial that tested triglyceride responses to a high-fat milkshake (83% fat and 700 kcal/m^2^) before and after three weeks of daily treatment with 160 mg of micronized fenofibrate.

Habitual dietary intake was assessed with the National Cancer Institute diet history questionnaire (DHQ) [8] while data on medical history, physical activity and other lifestyle variables such as alcohol intake were collected using an interviewer-administered questionnaire.

### Inflammatory markers

All inflammatory markers were measured in a central laboratory. hsCRP was measured on the Hitachi 911 analyzer using a latex particle enhanced immunoturbidimetric assay (Kamiya Biomedical Company, Seattle, WA). Other inflammatory markers including monocyte chemoattractant protein-1 (MCP1), interleukin-2 (IL2) soluble receptor-α, IL6 and TNF-α) were measured before and after treatment with fenofibrate using ELISA methods (R&D Systems Inc. Minneapolis, MN) as described elsewhere [9]. Quality assurance measurements for these assays were excellent and have been reported previously [10]. Briefly, the reliability coefficients for hsCRP, MCP-1, IL2 receptor-α, IL6 and TNF-α were 0.99, 0.91, 0.97, 0.92 and 0.76, respectively.

### Genotyping for TCF7L2 rs7903146 and rs12255372 polymorphisms

DNA isolation, genotyping procedure and quality control measures for rs7903146 and rs12255372 polymorphisms have been reported [11]. Genotyping was successful in 99.7% of the samples for rs7903146 and in the whole study population for rs12255372.

This study was approved by the institutional review boards at the University of Alabama at Birmingham, University of Minnesota, University of Utah and Tufts University.

### Statistical analysis

SAS Software version 9.1.3 (SAS Institute, Inc., Cary, NC) was used for statistical analyses. From the 1328 men and women in the GOLDN study, we excluded all subjects with a self-reported history of kidney disease, Grave's disease, and those with missing data on major exposure variables and potential confounders. The final data set consists of 1025 subjects in 186 families that have complete baseline measurements on inflammatory markers. In this subset, 776 men and women completed the fenofibrate trial and have complete data on both baseline and post-fenofibrate measurements of inflammatory markers and genotypes for rs7903146 polymorphism.

A subsample of unrelated subjects was used to test TCF7L2 genotypes for departure from Hardy-Weinberg Equilibrium. As an additional quality control measure we performed Mendelian checks on the genotype data. Variables were checked for normality and where necessary they were log- or square-root transformed. The significance of differences in the distribution of inflammatory markers and potential confounders by the TCF7L2 genotypes were tested using an ANOVA model using the Mixed procedure in SAS. This model adjusted for age, sex, field center, smoking, physical activity, alcohol intake and pedigree. Pedigree was included as a random effect to adjust for familial relationships. Habitual diet, BMI and waist circumference were also tested as potential confounders or intermediate variables in the TCF7L2-inflammation pathway. We further tested whether there were significant interactions between dietary variables and TCF7L2 polymorphisms and inflammatory markers.

## Results

The frequencies for the CC, CT and TT genotypes in rs7903146 polymorphism were 0.50, 0.39 and 0.11, respectively while they were 0.51, 0.39 and 0.11 for the GG, GT and TT genotypes in rs12255372 polymorphism, respectively. As showed previously in this [11] and other studies [2], the two polymorphisms (rs7903146 and rs12255372) are in strong linkage disequilibrium (*D' *= 0.9, *r *= 0.9). Thus the characteristics of the study population are shown only by rs7903146 polymorphism (Table 1). There were not significant differences (*P *> 0.05) across genotypes with regard to age, BMI, habitual diet, etc.

**Table 1:** Characteristics of the study population by *TCF7L2 *polymorphism (rs7903146)

	***TCF7L2 *genotypes for rs7903146**		
**Variable**	**CC**	**CT**	**TT**

n	514	398	113
Age (years)	48.7 (16.2)	48.8 (16.4)	48.5 (16.0)
Derived BMI (kg/m^2^)	28.4 (5.9)	28.3 (5.6)	28.0 (5.1)
Waist circumference (m)	96.1 (16.1)	97.2 (17.0)	96.7 (15.1)
Average systolic (mmHg)	114.4 (16.1)	116.1(17.0)	116.0 (16.4)
Average diastolic (mmHg)	67.6 (9.4)	68.6 (9.3)	68.7 (9.5)
Total dietary fat (% energy/d)	35.4 (7.0)	35.5 (6.3)	35.6 (7.1)
Dietary sat fat (% energy/d)	11.8 (2.7)	11.9 (2.6)	12.0 (2.9)
Dietary MUFA (% energy/d)	13.3 (2.9)	13.3 (2.6)	13.4 (2.8)
Dietary PUFA (% energy/d)	7.6 (2.2)	7.7 (2.0)	7.5 (2.2)
Dietary trans (% energy/d)	2.1 (0.6)	2.2 (0.6)	2.2 (0.7)
Dietary carbohydrate (% energy/d)	49.1 (8.5)	49.0 (7.9)	48.3 (9.2)
Dietary protein (% energy/d)	15.9 (2.9)	15.8 (2.7)	15.4 (3.2)
Caffeine (mg/d)	278 (439)	272 (413)	264 (380)

*P *> 0.05; *TCF7L2 *polymorphism rs7903146 is in strong LD (*D' *= 0.9) with rs12255372.

The distribution of inflammatory markers by TCF7L2 genotypes is shown in Table 2. In an additive model, there were no significant differences (*P *> 0.05) in the inflammatory markers (CRP, IL-2, IL-6, TNF-α and MCP-1) across TCF7L2 genotypes in the period before or after treatment with fenofibrate. Further adjustment for waist circumference or use of statins or other lipid-lowering drugs 4 weeks prior to the study did not change the significance of the associations.

**Table 2:** Concentrations of inflammatory markers by polymorphisms in the TCF7L2 gene^a^

**Variable**	**rs12255372 (G>T)**			**rs7903146 (C>T)**		
	
**Before fenofibrate**	**GG**	**GT**	**TT**	**CC**	**CT**	**TT**
n	512	390	115	514	398	113
CRP, mg/dL	0.23 (0.37)	0.25 (0.59)	0.33 (0.82)	0.23 (0.36)	0.25 (0.57)	0.31 (0.79)
IL-2 sR alpha, pg/mL	1014 (375)	1035 (353)	991 (332)	1021 (384)	1029 (350)	990 (324)
IL-6, pg/mL	2.04 (3.90)	1.88 (2.31)	1.88 (1.98)	2.10 (4.16)	1.74 (1.28)	2.00 (2.39)
TNF-alpha, pg/mL	3.25 (2.48)	3.73 (8.07)	3.35 (2.79)	3.28 (2.48)	3.68 (7.99)	3.36 (2.72)
MCP-1, pg/mL	208 (58)	212 (78)	205 (51)	209 (58)	210 (78)	207 (49)
**After fenofibrate**						
n	373	300	89	373	307	96
hsCRP, mg/dL	0.22 (0.35)	0.29 (0.53)	0.30 (0.61)	0.22 (0.37)	0.30 (0.54)	0.25 (0.50)
IL-2 sR alpha, pg/mL	1161 (539)	1186 (580)	1086 (354)	1169 (541)	1186 (573)	1063 (336)
IL-6, pg/mL	2.22 (4.23)	2.24 (2.68)	1.86 (2.08)	2.24 (4.34)	2.12 (2.21)	1.99 (2.19)
TNF-alpha, pg/mL	3.52 (2.36)	3.99 (5.99)	3.64 (2.53)	3.55 (2.36)	3.96 (5.93)	3.58 (2.46)
MCP-1, pg/mL	179 (94)	186 (102)	197 (80)	179 (93)	187 (103)	197 (77)

^a ^Values are means and SD; there were no significant differences across genotypes (*P *> 0.05).

We further tested for associations between genotypes and inflammatory markers using the dominant or recessive genetic model. No associations (*P *> 0.05) were detected under the dominant model. Under the recessive model we found that homozygote carriers of the T allele in either SNP had significantly higher (*P *< 0.05) post-fenofibrate concentrations of MCP-1 before and after adjusting for baseline MCP-1 concentrations, age, smoking, sex, field center, alcohol use, physical activity and familial relationships. The mean ± SD for the TT vs. CC+CT in rs7903146 were 197 ± 77 vs. 182 ± 98 pg/mL, respectively. The mean ± SD for the TT vs. GG+GT in rs12255372 were 197 ± 80 vs. 182 ± 98 pg/mL, respectively. No other significant associations were detected.

The difference in the concentrations of inflammatory markers (i.e., after treatment minus baseline measurement) also did not significantly differ (*P *> 0.05) by TCF7L2 genotypes (data not shown). These findings are not likely to be confounded by diet or other lifestyle variables since these variables did not differ across genotypes and did not significantly change the associations when added to the multivariate mixed models. Furthermore we did not detect any significant interactions between dietary variables and TCF7L2 polymorphisms (data not shown). The main dietary variables examined were intake of caffeine, total glycemic load, total carbohydrate, polyunsaturated fat, trans fat, saturated fat and total energy.

## Discussion

We have shown that TCF7L2 polymorphisms (rs12255372 and rs7903146) that have been consistently associated with type 2 diabetes are not significantly (*P *> 0.05) associated with any of the inflammatory markers in analyses before treatment with fenofibrate. A part from MCP-1 concentrations which were significantly higher (*P *< 0.05) among homozygote carriers of the T-allele in either SNP, there were no other significant associations observed for inflammatory markers measured after treatment with fenofibrate. These data suggest that the effects of TCF7L2 on diabetes may not be via inflammation. Although the findings from this study show a null association between TCF7L2 polymorphisms and inflammatory markers, they are important because there are no published studies on the association between TCF7L2 polymorphisms and inflammatory markers. These data support earlier studies that suggested impaired insulin secretion but not insulin sensitivity [12] as the main causal pathway for the relation between TCF7L2 gene and risk for type 2 diabetes [13].

## Abbreviations

TCF7L2: Transcription factor 7 like-2; hsCRP: High sensitivity C-reactive protein; IL2: Interleukin-2; IL6: Interleukin-6; TNF-α: Tumor necrosis factor-α; MCP: Macrophage chemoattractant protein-1; GOLDN: Genetics of Lipid-Lowering Drugs and Diet Network.

## Competing interests

The authors declare that they have no competing interests.

## Authors' contributions

EKK analyzed the data and drafted the manuscript. JMO and DW contributed toward genotyping for TCF7L2 SNPs. DKA, MYT, PNH, IBB and SPG designed the study and collected the data. All authors contributed to the scientific content, edited and approved the manuscript.
